# Supplementary figures and images for: Combined effect of physico-chemical and microbial quality of breeding habitat water on oviposition of malarial vector Anopheles subpictus
Source: PLoS One. 2023 Mar 10;18(3):e0282825. doi: 10.1371/journal.pone.0282825 (PMC10004544; doi:10.1371/journal.pone.0282825)

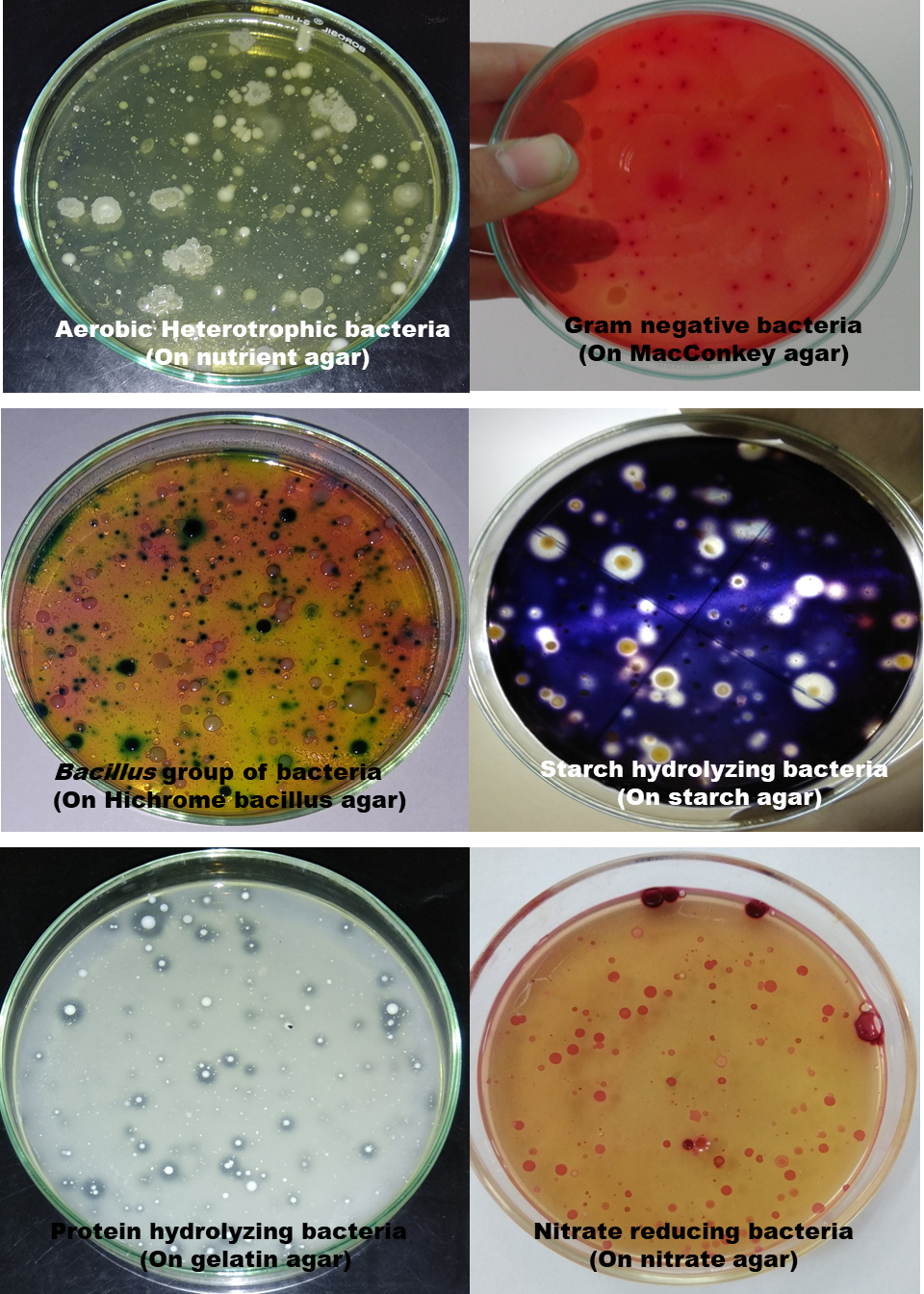

Supplement: S1 Fig — (TIF) [file pone.0282825.s004.tif]

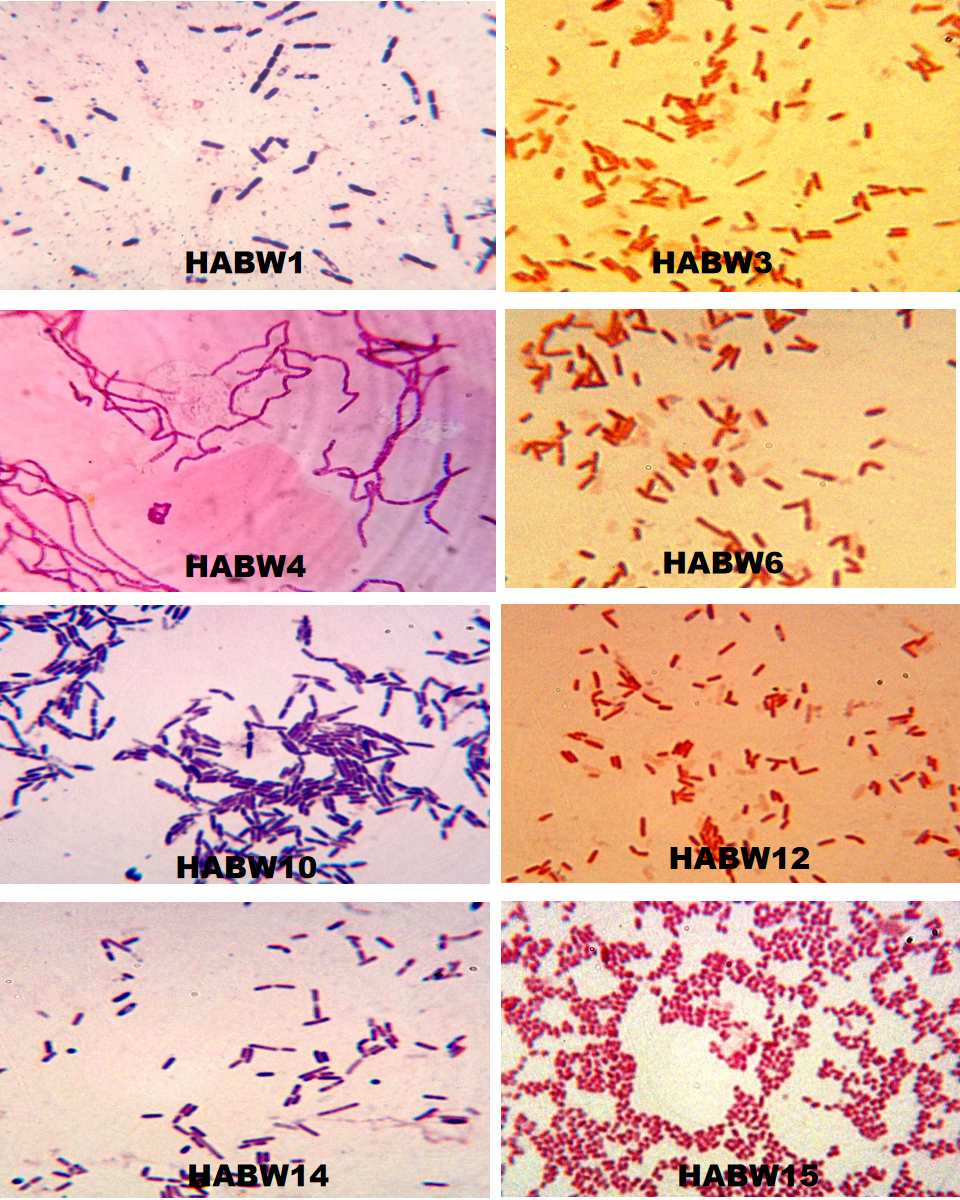

Supplement: S2 Fig — (TIF) [file pone.0282825.s005.tif]
