# Supplementary material for: Combined effect of physico-chemical and microbial quality of breeding habitat water on oviposition of malarial vector Anopheles subpictus
Source: PLoS One. 2023 Mar 10;18(3):e0282825. doi: 10.1371/journal.pone.0282825 (PMC10004544; doi:10.1371/journal.pone.0282825)
Supplement: S1 Table — (DOCX) [file pone.0282825.s006.docx]

**S1 Table: Friedman test for significant effect of season and habitat types on larval density of *Anopheles subpictus***

**A**

| **Ranks** | |
| --- | --- |
| Season | Mean Rank |
| Summer | 2.33 |
| Monsoon | 3.34 |
| Post-monsoon | 3.00 |
| Winter | 1.32 |

**B**

| **Test Statistics^a^** | |
| --- | --- |
| N | 60 |
| Chi-Square | 88.910 |
| df | 3 |
| Asymp. Sig. | .000 |
| a. Friedman Test | |
