# Supplementary material for: Combined effect of physico-chemical and microbial quality of breeding habitat water on oviposition of malarial vector Anopheles subpictus
Source: PLoS One. 2023 Mar 10;18(3):e0282825. doi: 10.1371/journal.pone.0282825 (PMC10004544; doi:10.1371/journal.pone.0282825)
Supplement: S2 Table — (DOCX) [file pone.0282825.s007.docx]

**S2 Table. One-Way ANOVA for physico-chemical parameters of different habitat types (ponds, drains & rice-fields) during summer season.**

| **One-Way ANOVA** | | | | | | |
| --- | --- | --- | --- | --- | --- | --- |
| **Parameter** | **DF** | **SS** | **MS** | **F (DFn, DFd)** | **P Value** | **Significance** |
| **Temperature** | **2** | 10.23 | 5.116 | F (2, 57) = 5.808 | 0.0051 | **Yes** |
| **pH** | 2 | 10.21 | 5.103 | F (2, 57) = 168.0 | <0.0001 | **Yes** |
| **Alkalinity** | 2 | 262993 | 131497 | F (2, 57) = 322.0 | <0.0001 | **Yes** |
| **DO** | 2 | 63.85 | 31.93 | F (2, 57) = 97.07 | <0.0001 | **Yes** |
| **Conductivity** | 2 | 70486 | 35243 | F (2, 57) = 5.835 | 0.0050 | **Yes** |
| **Hardness** | 2 | 201716 | 100858 | F (2, 57) = 29.58 | <0.0001 | **Yes** |
| **TDS** | 2 | 777176 | 388588 | F (2, 57) = 48.52 | <0.0001 | **Yes** |
| **Turbidity** | 2 | 394.5 | 197.3 | F (2, 57) = 126.0 | <0.0001 | **Yes** |
| **Chloride** | 2 | 347.6 | 173.8 | F (2, 57) = 1.933 | 0.1541 | No |
| **Phosphate** | 2 | 169.3 | 84.67 | F (2, 57) = 52.58 | <0.0001 | **Yes** |
| **Nitrate** | 2 | 103.1 | 51.53 | F (2, 57) = 57.66 | <0.0001 | **Yes** |
