# Supplementary material for: Combined effect of physico-chemical and microbial quality of breeding habitat water on oviposition of malarial vector Anopheles subpictus
Source: PLoS One. 2023 Mar 10;18(3):e0282825. doi: 10.1371/journal.pone.0282825 (PMC10004544; doi:10.1371/journal.pone.0282825)
Supplement: S3 Table — (DOCX) [file pone.0282825.s008.docx]

**S3 Table. One-Way ANOVA for physico-chemical parameters of different habitat types (ponds, drains & rice-fields) during monsoon season.**

| **One-Way ANOVA** | | | | | | |
| --- | --- | --- | --- | --- | --- | --- |
| **Parameter** | **DF** | **SS** | **MS** | **F (DFn, DFd)** | **P Value** | **Significance** |
| **Temperature** | **2** | 1.689 | 0.8447 | F (2, 57) = 1.406 | 0.2536 | No |
| **pH** | 2 | 13.53 | 6.765 | F (2, 57) = 56.09 | <0.0001 | **Yes** |
| **Alkalinity** | 2 | 118546 | 59273 | F (2, 57) = 48.31 | <0.0001 | **Yes** |
| **DO** | 2 | 152.6 | 76.29 | F (2, 57) = 135.3 | <0.0001 | **Yes** |
| **Conductivity** | 2 | 424740 | 212370 | F (2, 57) = 47.11 | <0.0001 | **Yes** |
| **Hardness** | 2 | 1029199 | 514600 | F (2, 57) = 98.51 | <0.0001 | **Yes** |
| **TDS** | 2 | 469776 | 234888 | F (2, 57) = 39.37 | <0.0001 | **Yes** |
| **Turbidity** | 2 | 610.9 | 305.4 | F (2, 57) = 180.9 | <0.0001 | **Yes** |
| **Chloride** | 2 | 97.63 | 48.81 | F (2, 57) = 0.8731 | 0.4232 | No |
| **Phosphate** | 2 | 56.08 | 28.04 | F (2, 57) = 32.03 | <0.0001 | **Yes** |
| **Nitrate** | 2 | 83.66 | 41.83 | F (2, 57) = 45.62 | <0.0001 | **Yes** |
