# Supplementary material for: Combined effect of physico-chemical and microbial quality of breeding habitat water on oviposition of malarial vector Anopheles subpictus
Source: PLoS One. 2023 Mar 10;18(3):e0282825. doi: 10.1371/journal.pone.0282825 (PMC10004544; doi:10.1371/journal.pone.0282825)
Supplement: S4 Table — (DOCX) [file pone.0282825.s009.docx]

**S4 Table. One-Way ANOVA for physico-chemical parameters of different habitat types (ponds, drains & rice-fields) during post-monsoon season.**

| **One-Way ANOVA** | | | | | | |
| --- | --- | --- | --- | --- | --- | --- |
| **Parameter** | **DF** | **SS** | **MS** | **F (DFn, DFd)** | **P Value** | **Significance** |
| **Temperature** | 2 | 9.977 | 4.989 | F (2, 57) = 7.737 | 0.0011 | **Yes** |
| **pH** | 2 | 6.811 | 3.406 | F (2, 57) = 94.46 | <0.0001 | **Yes** |
| **Alkalinity** | 2 | 189631 | 94815 | F (2, 57) = 306.7 | <0.0001 | **Yes** |
| **DO** | 2 | 110.3 | 55.17 | F (2, 57) = 202.7 | <0.0001 | **Yes** |
| **Conductivity** | 2 | 333611 | 166806 | F (2, 57) = 30.04 | <0.0001 | **Yes** |
| **Hardness** | 2 | 970123 | 485061 | F (2, 57) = 113.1 | <0.0001 | **Yes** |
| **TDS** | 2 | 317248 | 158624 | F (2, 57) = 50.95 | <0.0001 | **Yes** |
| **Turbidity** | 2 | 660.8 | 330.4 | F (2, 57) = 206.4 | <0.0001 | **Yes** |
| **Chloride** | 2 | 50.34 | 25.17 | F (2, 57) = 0.1750 | 0.8399 | No |
| **Phosphate** | 2 | 70.27 | 35.13 | F (2, 57) = 34.01 | <0.0001 | **Yes** |
| **Nitrate** | 2 | 141.6 | 70.81 | F (2, 57) = 113.3 | <0.0001 | **Yes** |
