# Supplementary material for: Combined effect of physico-chemical and microbial quality of breeding habitat water on oviposition of malarial vector Anopheles subpictus
Source: PLoS One. 2023 Mar 10;18(3):e0282825. doi: 10.1371/journal.pone.0282825 (PMC10004544; doi:10.1371/journal.pone.0282825)
Supplement: S5 Table — (DOCX) [file pone.0282825.s010.docx]

**S5 Table. Mann-Whitney test for physico-chemical parameters between ponds and drains during winter season.**

| **Parameter** | **U-value** | **P Value** | **Significance** |
| --- | --- | --- | --- |
| **Temperature** | 92.50 | 0.0029 | **Yes** |
| **pH** | 56.50 | <0.0001 | **Yes** |
| **Alkalinity** | 1 | <0.0001 | **Yes** |
| **DO** | 0 | <0.0001 | **Yes** |
| **Conductivity** | 66 | 0.0002 | **Yes** |
| **Hardness** | 19 | <0.0001 | **Yes** |
| **TDS** | 22 | <0.0001 | **Yes** |
| **Turbidity** | 0 | <0.0001 | **Yes** |
| **Chloride** | 185 | 0.6980 | No |
| **Phosphate** | 0 | <0.0001 | **Yes** |
| **Nitrate** | 10 | <0.0001 | **Yes** |
