# Supplementary material for: Combined effect of physico-chemical and microbial quality of breeding habitat water on oviposition of malarial vector Anopheles subpictus
Source: PLoS One. 2023 Mar 10;18(3):e0282825. doi: 10.1371/journal.pone.0282825 (PMC10004544; doi:10.1371/journal.pone.0282825)
Supplement: S6 Table — (DOCX) [file pone.0282825.s011.docx]

**S6 Table: Principal Component Analysis (PCA) for larval density and physico-chemical parameters of habitat water during summer season.**

**A**

| **Eigenvalues:** | | | | | | | | | | | | |
| --- | --- | --- | --- | --- | --- | --- | --- | --- | --- | --- | --- | --- |
|  | **F1** | **F2** | **F3** | **F4** | **F5** | **F6** | **F7** | **F8** | **F9** | **F10** | **F11** | **F12** |
| Eigenvalue | 6.759 | 1.773 | 1.155 | 0.667 | 0.498 | 0.283 | 0.267 | 0.231 | 0.146 | 0.128 | 0.053 | 0.041 |
| Variability (%) | 56.326 | 14.775 | 9.621 | 5.555 | 4.147 | 2.362 | 2.223 | 1.929 | 1.213 | 1.066 | 0.442 | 0.341 |
| Cumulative % | 56.326 | 71.101 | 80.722 | 86.277 | 90.425 | 92.787 | 95.010 | 96.939 | 98.151 | 99.217 | 99.659 | 100.000 |

**B**

| **Correlations between variables and factors:** | | | | | |
| --- | --- | --- | --- | --- | --- |
|  | **F1** | **F2** | **F3** | **F4** | **F5** |
| L.D | -0.651 | 0.593 | 0.264 | -0.251 | -0.190 |
| Temperature | -0.022 | 0.242 | 0.892 | 0.343 | 0.154 |
| pH | 0.815 | -0.487 | 0.044 | 0.126 | 0.012 |
| Alkalinity | 0.865 | -0.342 | 0.043 | 0.098 | -0.131 |
| D.O | -0.931 | 0.019 | -0.130 | -0.155 | -0.001 |
| Conductivity | 0.604 | 0.472 | -0.292 | 0.025 | 0.542 |
| Hardness | 0.820 | 0.281 | 0.029 | -0.269 | 0.040 |
| TDS | 0.754 | 0.521 | 0.109 | -0.230 | -0.112 |
| Turbidity | 0.946 | 0.050 | 0.042 | -0.057 | 0.003 |
| Chloride | 0.282 | 0.636 | -0.385 | 0.523 | -0.284 |
| Phosphate | 0.839 | -0.139 | 0.143 | -0.170 | -0.138 |
| Nitrate | 0.878 | 0.095 | 0.020 | -0.068 | -0.114 |

**C**

| **Contribution of the variables (%):** | | | |  |  |
| --- | --- | --- | --- | --- | --- |
|  | **F1** | **F2** | **F3** | **F4** | **F5** |
| L.D | 6.274 | 19.830 | 6.035 | 9.453 | 7.263 |
| Temperature | 0.007 | 3.310 | 68.879 | 17.650 | 4.744 |
| pH | 9.822 | 13.376 | 0.169 | 2.377 | 0.029 |
| Alkalinity | 11.083 | 6.602 | 0.162 | 1.440 | 3.456 |
| D.O | 12.814 | 0.020 | 1.470 | 3.586 | 0.000 |
| Conductivity | 5.401 | 12.582 | 7.406 | 0.092 | 58.962 |
| Hardness | 9.942 | 4.444 | 0.073 | 10.863 | 0.319 |
| TDS | 8.408 | 15.313 | 1.023 | 7.922 | 2.511 |
| Turbidity | 13.250 | 0.141 | 0.150 | 0.481 | 0.002 |
| Chloride | 1.180 | 22.791 | 12.833 | 41.088 | 16.245 |
| Phosphate | 10.403 | 1.084 | 1.766 | 4.358 | 3.839 |
| Nitrate | 11.415 | 0.505 | 0.035 | 0.690 | 2.629 |
