# Supplementary material for: Combined effect of physico-chemical and microbial quality of breeding habitat water on oviposition of malarial vector Anopheles subpictus
Source: PLoS One. 2023 Mar 10;18(3):e0282825. doi: 10.1371/journal.pone.0282825 (PMC10004544; doi:10.1371/journal.pone.0282825)
Supplement: S7 Table — (DOCX) [file pone.0282825.s012.docx]

| **Eigenvalues:** | | | | | | | | | | | | |
| --- | --- | --- | --- | --- | --- | --- | --- | --- | --- | --- | --- | --- |
|  | **F1** | **F2** | **F3** | **F4** | **F5** | **F6** | **F7** | **F8** | **F9** | **F10** | **F11** | **F12** |
| **Eigenvalue** | 7.263 | 1.443 | 1.074 | 0.787 | 0.391 | 0.340 | 0.247 | 0.170 | 0.145 | 0.079 | 0.038 | 0.023 |
| **Variability (%)** | 60.527 | 12.025 | 8.954 | 6.555 | 3.261 | 2.836 | 2.057 | 1.413 | 1.210 | 0.660 | 0.314 | 0.189 |
| **Cumulative %** | 60.527 | 72.551 | 81.505 | 88.059 | 91.321 | 94.156 | 96.213 | 97.626 | 98.837 | 99.497 | 99.811 | 100.000 |

**S7 Table: Principal Component Analysis (PCA) for larval density and physico-chemical parameters of habitat water during monsoon season.**

**A**

**B**

| **Correlations between variables and factors:** | | | | | |
| --- | --- | --- | --- | --- | --- |
|  | **F1** | **F2** | **F3** | **F4** | **F5** |
| L.D | -0.551 | -0.679 | 0.002 | 0.362 | 0.045 |
| Temperature | 0.113 | 0.704 | 0.398 | 0.540 | 0.157 |
| pH | 0.873 | -0.027 | -0.057 | -0.143 | -0.241 |
| Alkalinity | 0.849 | 0.362 | 0.055 | -0.163 | -0.168 |
| D.O | -0.931 | -0.147 | 0.062 | -0.117 | 0.152 |
| Conductivity | 0.751 | -0.430 | 0.152 | 0.336 | -0.155 |
| Hardness | 0.941 | -0.223 | 0.056 | 0.161 | 0.042 |
| TDS | 0.848 | -0.145 | 0.106 | -0.182 | 0.319 |
| Turbidity | 0.952 | -0.081 | -0.056 | 0.147 | -0.102 |
| Chloride | -0.060 | -0.234 | 0.924 | -0.268 | -0.066 |
| Phosphate | 0.867 | -0.070 | -0.063 | -0.186 | 0.289 |
| Nitrate | 0.875 | -0.108 | -0.088 | 0.055 | 0.171 |

**C**

| **Contribution of the variables (%):** | | | | | |
| --- | --- | --- | --- | --- | --- |
|  | **F1** | **F2** | **F3** | **F4** | **F5** |
| L.D | 4.187 | 31.945 | 0.000 | 16.684 | 0.511 |
| Temperature | 0.175 | 34.303 | 14.758 | 37.124 | 6.291 |
| pH | 10.487 | 0.050 | 0.299 | 2.596 | 14.883 |
| Alkalinity | 9.923 | 9.068 | 0.283 | 3.365 | 7.170 |
| D.O | 11.945 | 1.501 | 0.354 | 1.741 | 5.870 |
| Conductivity | 7.755 | 12.821 | 2.161 | 14.356 | 6.175 |
| Hardness | 12.187 | 3.461 | 0.295 | 3.282 | 0.454 |
| TDS | 9.904 | 1.448 | 1.048 | 4.199 | 26.007 |
| Turbidity | 12.480 | 0.459 | 0.294 | 2.762 | 2.663 |
| Chloride | 0.050 | 3.792 | 79.418 | 9.122 | 1.127 |
| Phosphate | 10.359 | 0.340 | 0.369 | 4.385 | 21.360 |
| Nitrate | 10.549 | 0.810 | 0.721 | 0.384 | 7.489 |
