# Supplementary material for: Combined effect of physico-chemical and microbial quality of breeding habitat water on oviposition of malarial vector Anopheles subpictus
Source: PLoS One. 2023 Mar 10;18(3):e0282825. doi: 10.1371/journal.pone.0282825 (PMC10004544; doi:10.1371/journal.pone.0282825)
Supplement: S8 Table — (DOCX) [file pone.0282825.s013.docx]

**S8 Table: Principal Component Analysis (PCA) for larval density and physico-chemical parameters of habitat water during post-monsoon season.**

**A**

| **Eigenvalues:** | | | | | | | | | | | | |
| --- | --- | --- | --- | --- | --- | --- | --- | --- | --- | --- | --- | --- |
|  | **F1** | **F2** | **F3** | **F4** | **F5** | **F6** | **F7** | **F8** | **F9** | **F10** | **F11** | **F12** |
| Eigenvalue | 7.271 | 1.589 | 1.190 | 0.772 | 0.359 | 0.282 | 0.187 | 0.141 | 0.087 | 0.068 | 0.030 | 0.025 |
| Variability (%) | 60.594 | 13.238 | 9.915 | 6.430 | 2.988 | 2.352 | 1.559 | 1.175 | 0.722 | 0.564 | 0.251 | 0.211 |
| Cumulative % | 60.594 | 73.833 | 83.748 | 90.179 | 93.166 | 95.518 | 97.078 | 98.252 | 98.974 | 99.538 | 99.789 | 100.000 |

**B**

| **Correlations between variables and factors:** | | | | | |
| --- | --- | --- | --- | --- | --- |
|  | **F1** | **F2** | **F3** | **F4** | **F5** |
| LD | -0.621 | 0.688 | -0.144 | 0.172 | -0.048 |
| Temperature | -0.348 | -0.471 | 0.405 | 0.696 | -0.003 |
| pH | 0.845 | -0.334 | -0.113 | 0.022 | -0.021 |
| Alkalinity | 0.738 | -0.611 | 0.029 | -0.185 | -0.029 |
| DO | -0.920 | 0.029 | 0.169 | -0.021 | 0.035 |
| Conductivity | 0.845 | 0.076 | 0.373 | -0.098 | -0.237 |
| Hardness | 0.856 | 0.440 | -0.037 | 0.117 | -0.048 |
| TDS | 0.820 | 0.175 | -0.204 | 0.328 | -0.253 |
| Turbidity | 0.943 | 0.106 | -0.169 | 0.096 | -0.072 |
| Chloride | 0.307 | 0.323 | 0.855 | -0.179 | -0.036 |
| Phosphate | 0.831 | 0.245 | 0.135 | 0.215 | 0.403 |
| Nitrate | 0.931 | 0.040 | -0.054 | -0.057 | 0.250 |

**C**

| **Contribution of the variables (%):** | | | | | |
| --- | --- | --- | --- | --- | --- |
|  | **F1** | **F2** | **F3** | **F4** | **F5** |
| LD | 5.300 | 29.829 | 1.748 | 3.845 | 0.651 |
| Temperature | 1.663 | 13.983 | 13.779 | 62.779 | 0.003 |
| pH | 9.824 | 7.020 | 1.076 | 0.065 | 0.118 |
| Alkalinity | 7.496 | 23.521 | 0.070 | 4.454 | 0.234 |
| DO | 11.630 | 0.053 | 2.413 | 0.057 | 0.340 |
| Conductivity | 9.812 | 0.360 | 11.714 | 1.254 | 15.677 |
| Hardness | 10.076 | 12.163 | 0.117 | 1.787 | 0.651 |
| TDS | 9.255 | 1.922 | 3.498 | 13.962 | 17.811 |
| Turbidity | 12.240 | 0.705 | 2.414 | 1.198 | 1.441 |
| Chloride | 1.299 | 6.556 | 61.387 | 4.174 | 0.369 |
| Phosphate | 9.497 | 3.787 | 1.541 | 5.997 | 45.205 |
| Nitrate | 11.908 | 0.101 | 0.242 | 0.428 | 17.498 |
