# Supplementary material for: Combined effect of physico-chemical and microbial quality of breeding habitat water on oviposition of malarial vector Anopheles subpictus
Source: PLoS One. 2023 Mar 10;18(3):e0282825. doi: 10.1371/journal.pone.0282825 (PMC10004544; doi:10.1371/journal.pone.0282825)
Supplement: S9 Table — (DOCX) [file pone.0282825.s014.docx]

**S9 Table: Principal Component Analysis (PCA) for larval density and physico-chemical parameters of habitat water during winter season.**

**A**

| **Eigenvalues:** | | | | | | | | | | | | |
| --- | --- | --- | --- | --- | --- | --- | --- | --- | --- | --- | --- | --- |
|  | **F1** | **F2** | **F3** | **F4** | **F5** | **F6** | **F7** | **F8** | **F9** | **F10** | **F11** | **F12** |
| Eigenvalue | 7.316 | 1.292 | 0.981 | 0.658 | 0.485 | 0.447 | 0.352 | 0.199 | 0.103 | 0.078 | 0.047 | 0.043 |
| Variability (%) | 60.964 | 10.768 | 8.174 | 5.481 | 4.040 | 3.726 | 2.932 | 1.658 | 0.862 | 0.647 | 0.392 | 0.356 |
| Cumulative % | 60.964 | 71.732 | 79.906 | 85.387 | 89.427 | 93.153 | 96.085 | 97.744 | 98.605 | 99.253 | 99.644 | 100.000 |

**B**

| **Correlations between variables and factors:** | | | | | |
| --- | --- | --- | --- | --- | --- |
|  | **F1** | **F2** | **F3** | **F4** | **F5** |
| L.D | -0.892 | 0.252 | 0.008 | -0.064 | 0.127 |
| Temperature | -0.483 | -0.613 | 0.509 | -0.045 | 0.020 |
| pH | 0.753 | -0.052 | 0.210 | -0.343 | 0.472 |
| Alkalinity | 0.939 | -0.089 | 0.149 | -0.179 | 0.030 |
| D.O | -0.924 | 0.234 | -0.090 | 0.037 | 0.021 |
| Conductivity | 0.663 | 0.231 | 0.303 | 0.526 | 0.252 |
| Hardness | 0.839 | 0.117 | -0.242 | 0.304 | 0.102 |
| TDS | 0.747 | 0.047 | 0.380 | 0.028 | -0.356 |
| Turbidity | 0.907 | -0.169 | 0.076 | 0.106 | -0.162 |
| Chloride | 0.150 | 0.827 | 0.381 | -0.236 | -0.100 |
| Phosphate | 0.850 | 0.063 | -0.347 | -0.157 | 0.007 |
| Nitrate | 0.846 | -0.052 | -0.284 | -0.199 | -0.084 |

**C**

| **Contribution of the variables (%):** | | | | | |
| --- | --- | --- | --- | --- | --- |
|  | **F1** | **F2** | **F3** | **F4** | **F5** |
| L.D | 10.876 | 4.908 | 0.007 | 0.615 | 3.348 |
| Temperature | 3.188 | 29.036 | 26.425 | 0.308 | 0.084 |
| pH | 7.750 | 0.212 | 4.495 | 17.932 | 45.909 |
| Alkalinity | 12.045 | 0.609 | 2.258 | 4.860 | 0.180 |
| D.O | 11.663 | 4.246 | 0.825 | 0.213 | 0.092 |
| Conductivity | 6.012 | 4.145 | 9.347 | 41.985 | 13.136 |
| Hardness | 9.619 | 1.054 | 5.986 | 14.012 | 2.134 |
| TDS | 7.626 | 0.172 | 14.760 | 0.122 | 26.194 |
| Turbidity | 11.241 | 2.210 | 0.582 | 1.702 | 5.385 |
| Chloride | 0.309 | 52.890 | 14.830 | 8.456 | 2.073 |
| Phosphate | 9.878 | 0.310 | 12.270 | 3.764 | 0.011 |
| Nitrate | 9.792 | 0.206 | 8.215 | 6.031 | 1.456 |
