# Supplementary material for: Combined effect of physico-chemical and microbial quality of breeding habitat water on oviposition of malarial vector Anopheles subpictus
Source: PLoS One. 2023 Mar 10;18(3):e0282825. doi: 10.1371/journal.pone.0282825 (PMC10004544; doi:10.1371/journal.pone.0282825)
Supplement: S10 Table — (DOCX) [file pone.0282825.s015.docx]

**Table S10: Univariate Tests of Significance for larval density (L.D)**

**A**

| Effect | Sigma-restricted parameterization Effective hypothesis decomposition; Std. Error of Estimate: 4.001217 | | | | |
| --- | --- | --- | --- | --- | --- |
|  | \| SS \| \| --- \| | \| Degree of Freedom \| \| --- \| | \| MS \| \| --- \| | \| F \| \| --- \| | \| p \| \| --- \| |
| \| Intercept \| \| --- \| | **97.119** | **1** | **97.119** | **6.06623** | **0.014592** |
| \| Temperature \| \| --- \| | 28.395 | 1 | 28.395 | 1.77361 | 0.184394 |
| \| **pH** \| \| --- \| | **89.774** | **1** | **89.774** | **5.60744** | **0.018801** |
| \| **Alkalinity** \| \| --- \| | **1160.285** | **1** | **1160.285** | **72.47369** | **0.000000** |
| \| **D.O** \| \| --- \| | **427.596** | **1** | **427.596** | **26.70846** | **0.000001** |
| \| Conductivity \| \| --- \| | 2.055 | 1 | 2.055 | 0.12837 | 0.720496 |
| \| Hardness \| \| --- \| | 24.237 | 1 | 24.237 | 1.51391 | 0.219934 |
| \| TDS \| \| --- \| | 18.123 | 1 | 18.123 | 1.13199 | 0.288584 |
| \| Turbidity \| \| --- \| | 57.564 | 1 | 57.564 | 3.59559 | 0.059319 |
| \| Chloride \| \| --- \| | 3.220 | 1 | 3.220 | 0.20116 | 0.654255 |
| \| Phosphate \| \| --- \| | 26.805 | 1 | 26.805 | 1.67430 | 0.197119 |
| \| Nitrate \| \| --- \| | 0.317 | 1 | 0.317 | 0.01982 | 0.888184 |
| \| Error \| \| --- \| | 3330.027 | 208 | 16.010 |  |  |

Parameters in ‘bold’ indicates significant effect on larval density.

**B**

| Dependent Variable | Test of SS Whole Model vs. SS Residual | | | | | | | | | | |
| --- | --- | --- | --- | --- | --- | --- | --- | --- | --- | --- | --- |
|  | \| Multiple R \| \| --- \| | \| Multiple R² \| \| --- \| | \| Adjusted R² \| \| --- \| | \| SS Model \| \| --- \| | \| df Model \| \| --- \| | \| MS Model \| \| --- \| | \| SS Residual \| \| --- \| | \| df Residual \| \| --- \| | \| MS Residual \| \| --- \| | \| F \| \| --- \| | \| p \| \| --- \| |
| \| L.D \| \| --- \| | 0.782835 | 0.612831 | 0.592356 | 5270.946 | 11 | 479.1769 | 3330.027 | 208 | 16.00974 | 29.93033 | 0.00 |

**C**

| Effect | Parameter Estimates: Sigma-restricted parameterization | | | | | | | | | |
| --- | --- | --- | --- | --- | --- | --- | --- | --- | --- | --- |
|  | \| L.D Param. \| \| --- \| | \| L.D Std.Err \| \| --- \| | \| L.D t \| \| --- \| | \| L.D p \| \| --- \| | \| -95.00% Cnf.Lmt \| \| --- \| | \| +95.00% Cnf.Lmt \| \| --- \| | \| L.D Beta (ß) \| \| --- \| | \| L.D St.Err.ß \| \| --- \| | \| -95.00% Cnf.Lmt \| \| --- \| | \| +95.00% Cnf.Lmt \| \| --- \| |
| \| Intercept \| \| --- \| | **-19.5339** | **7.931041** | **-2.46297** | **0.014592** | **-35.1695** | **-3.89840** |  |  |  |  |
| \| Temperature \| \| --- \| | 0.1307 | 0.098121 | 1.33177 | 0.184394 | -0.0628 | 0.32411 | 0.070309 | 0.052794 | -0.033770 | 0.174389 |
| \| pH \| \| --- \| | **2.0551** | **0.867847** | **2.36800** | **0.018801** | **0.3442** | **3.76597** | **0.181006** | **0.076438** | **0.030313** | **0.331698** |
| \| Alkalinity \| \| --- \| | **-0.0788** | **0.009261** | **-8.51315** | **0.000000** | **-0.0971** | **-0.06058** | **-0.771529** | **0.090628** | **-0.950197** | **-0.592862** |
| \| D.O \| \| --- \| | **2.3209** | **0.449091** | **5.16802** | **0.000001** | **1.4356** | **3.20626** | **0.560195** | **0.108396** | **0.346498** | **0.773891** |
| \| Conductivity \| \| --- \| | 0.0015 | 0.004233 | 0.35828 | 0.720496 | -0.0068 | 0.00986 | 0.026206 | 0.073144 | -0.117993 | 0.170405 |
| \| Hardness \| \| --- \| | 0.0062 | 0.005062 | 1.23041 | 0.219934 | -0.0038 | 0.01621 | 0.128630 | 0.104542 | -0.077469 | 0.334729 |
| \| TDS \| \| --- \| | 0.0033 | 0.003070 | 1.06395 | 0.288584 | -0.0028 | 0.00932 | 0.066523 | 0.062524 | -0.056740 | 0.189785 |
| \| Turbidity \| \| --- \| | 0.4488 | 0.236680 | 1.89620 | 0.059319 | -0.0178 | 0.91539 | 0.240678 | 0.126926 | -0.009549 | 0.490905 |
| \| Chloride \| \| --- \| | 0.0134 | 0.029808 | 0.44851 | 0.654255 | -0.0454 | 0.07213 | 0.023674 | 0.052783 | -0.080385 | 0.127732 |
| \| Phosphate \| \| --- \| | 0.3779 | 0.292055 | 1.29395 | 0.197119 | -0.1979 | 0.95367 | 0.099838 | 0.077158 | -0.052274 | 0.251950 |
| \| Nitrate \| \| --- \| | -0.0484 | 0.343477 | -0.14078 | 0.888184 | -0.7255 | 0.62879 | -0.012298 | 0.087357 | -0.184517 | 0.159921 |
