# Supplementary material for: Combined effect of physico-chemical and microbial quality of breeding habitat water on oviposition of malarial vector Anopheles subpictus
Source: PLoS One. 2023 Mar 10;18(3):e0282825. doi: 10.1371/journal.pone.0282825 (PMC10004544; doi:10.1371/journal.pone.0282825)
Supplement: Attachment — Submitted filename: Review comments-PONE-D-22-19958.docx [file pone.0282825.s017.docx]

Review comments on Manuscript entitled **“Combined effect of physico-chemical and microbial quality of breeding habitat water on oviposition of malarial vector Anopheles subpictus”.**

The authors have addressed most of the comments that were provided in the previous review, however, these are some few more comments that the author may consider to improve further the quality of the manuscript.

**Introduction:**

Line 48 – Malarial pathogens, change to “Malaria parasites”

Line 48 – This statement “they can easily transmit this protozoan parasite from one human host to another” is redundant, remove it. The remaining sentence should read “Females of different species of *Anopheles* mosquitoes serve as vectors of malaria parasites due to their blood sucking behavior”

Line 69 – 72: …where researchers showed….note that, researchers do not show, rather studies finds or show/indicates …

..led to reduce .. it should be “led to a reduction in”

The background has not adequately indicated as to why this study was conducted and what is the significance of the study. Authors should ensure that the rationale of the study is clearly stated in the study background.

It is also important to slightly describe the malaria burden in the district/area.

**Methodology**

**Study area and study period**

Based on the nature of the study, it is important to describe some basic climatic features of the study area, including rainfall, temperature, seasons of the year, etc.

**Field survey & collection of habitat water**

- Authors have to describe in detail how did they identify and select the breeding sites on which samples were collected. Was it throughout the district, were they randomly sampled, how many were they?

**Processing of water samples for bacterial isolation**

This subheading should be placed before **Analysis of bacterial populations of water**

**Results**

For clarity and easy comprehension re-arrange table 2 as shown below

**Table 2. Seasonwise physico-chemical parameters (Mean±S.E.) of different habitat** 299

**waterbodies of *Anopheles subpictus*.**

| **Parameter** | **Summer** | | | **Monsoon** | | | **Post-monsoon** | | | **Winter** | | |
| --- | --- | --- | --- | --- | --- | --- | --- | --- | --- | --- | --- | --- |
|  | **Pond** | **Dain** | **Rice field** | **Pond** | **Dain** | **Rice field** | **Pond** | **Dain** | **Rice field** | **Pond** | **Dain** | **Rice field** |
| Temperature (^o^C) |  |  |  |  |  |  |  |  |  |  |  |  |
| DO (mg/L |  |  |  |  |  |  |  |  |  |  |  |  |
| Alk |  |  |  |  |  |  |  |  |  |  |  |  |
| pH |  |  |  |  |  |  |  |  |  |  |  |  |
| Turb |  |  |  |  |  |  |  |  |  |  |  |  |
| TDS |  |  |  |  |  |  |  |  |  |  |  |  |
| T.H |  |  |  |  |  |  |  |  |  |  |  |  |
| E.C |  |  |  |  |  |  |  |  |  |  |  |  |
| Cl^-^ |  |  |  |  |  |  |  |  |  |  |  |  |
| NO_3_- |  |  |  |  |  |  |  |  |  |  |  |  |
| PO_4_- |  |  |  |  |  |  |  |  |  |  |  |  |
