# Supplementary material for: Combined effect of physico-chemical and microbial quality of breeding habitat water on oviposition of malarial vector Anopheles subpictus
Source: PLoS One. 2023 Mar 10;18(3):e0282825. doi: 10.1371/journal.pone.0282825 (PMC10004544; doi:10.1371/journal.pone.0282825)
Supplement: Attachment — Submitted filename: Response to reviewers comments.docx [file pone.0282825.s018.docx]

To

Cinzia Calvio

Academic Editor

PLoS ONE

**Sub:** Submission of revised manuscript (PONE-D-22-19958R1)

Dear Sir,

Thank you for your email dated 16/01/2023 enclosing the reviewers’ comments. We are very much thankful to you and to the hon’ble reviewers for their valuable comments and constructive suggestions. We have carefully reviewd all the comments and have revised the manuscript accordingly. Our responses are given point-by-point manner below and the modified portions have been highlighted in the revised manuscript.

We look forward to hearing from you in due course.

With regards,

Dr. Soumendranath Chatterjee

Professor

Department of Zoology

The University of Burdwan

Golapbag, Burdwan, West Bengal

- **Introduction:**
- **Comment 1:** Line 48 – Malarial pathogens, change to “Malaria parasites”.
- **Author’s response:** According to the suggestion of hon’ble reviewer in line 48 the words ‘Malarial pathogens’ have been changed to “Malaria parasites”.
- **Comment 2:** Line 48 – This statement “they can easily transmit this protozoan parasite from one human host to another” is redundant, remove it. The remaining sentence should read “Females of different species of *Anopheles* mosquitoes serve as vectors of malaria parasites due to their blood sucking behavior”.
- **Author’s response:** According to the suggestion of hon’ble reviewer, the statement “they can easily transmit this protozoan parasite from one human host to another” has been removed from the revised manuscript and written as “Females of different species of *Anopheles* mosquitoes serve as vectors of malaria parasites due to their blood sucking behavior” (Line no. 47-48).
- **Comment 3:** Line 69 – 72: …where researchers showed….note that, researchers do not show, rather studies finds or show/indicates …..led to reduce .. it should be “led to a reduction in”
- **Author’s response:** According to the suggestion of hon’ble reviewer, this sentence has been modified as “Several studies indicated that killing of these bacteria by sterilization technique or addition of effective antibiotics to the breeding habitat water led to a reduction in ovipositional response by adult gravid female mosquitoes” (Line no. 80-82 in the revised manuscript).
- **Comment 4:** The background has not adequately indicated as to why this study was conducted and what is the significance of the study. Authors should ensure that the rationale of the study is clearly stated in the study background.
- **Author’s response:** According to the suggestion of hon’ble reviewer, background of the study have been described in the revised manuscript (line no. 83-95).
- **Comment 4:** It is also important to slightly describe the malaria burden in the district/area.
- **Author’s response:** According to the suggestion of hon’ble reviewer, malaria burden in the district have been described in the revised manuscript (line no. 58-63).
- **Methodology**
- **Comment 5:** **Study area and study period:** Based on the nature of the study, it is important to describe some basic climatic features of the study area, including rainfall, temperature, seasons of the year, etc.
- **Author’s response:** According to the suggestion of hon’ble reviewer, climatic features of the study area have been described in the methodology section of the revised manuscript (line no. 102-104).
- **Comment 6:** **Field survey & collection of habitat water**

Authors have to describe in detail how did they identify and select the breeding sites on which samples were collected. Was it	throughout the district, were they randomly sampled, how many were they?

- **Author’s response:** Samples were collected from four blocks (Tarakeswar, Singur, Chinsurah-Mogra and Panduah) of Hooghly district (mentioned in materials and methodology section under study area and study period; line no. 100-102). In these four blocks of Hooghly district, suspected water bodies were checked randomly for the presence of *Anopheles subpictus* larvae. Samples were collected from those water bodies, where larval prevalence of *An. subpictus* were recorded during the study period (line no. 106-108).
- **Comment 7:** **Processing of water samples for bacterial isolation**

This subheading should be placed before **Analysis of bacterial populations of water**

- **Author’s response:** According to the suggestion of hon’ble reviewer, the subheading “**Processing of water samples for bacterial isolation”** (line no. 135) was added before **“Analysis of bacterial populations of water”**
- **Results**
- **Comment 8: For clarity and easy comprehension re-arrange table 2 as shown below**

**Table 2. Seasonwise physico-chemical parameters (Mean±S.E.) of different habitat waterbodies of *Anopheles subpictus*.**

| **Parameter** | **Summer** | | | **Monsoon** | | | **Post-monsoon** | | | **Winter** | | |
| --- | --- | --- | --- | --- | --- | --- | --- | --- | --- | --- | --- | --- |
|  | **Pond** | **Dain** | **Rice field** | **Pond** | **Dain** | **Rice field** | **Pond** | **Dain** | **Rice field** | **Pond** | **Dain** | **Rice field** |
| Temperature (^o^C) |  |  |  |  |  |  |  |  |  |  |  |  |
| DO (mg/L) |  |  |  |  |  |  |  |  |  |  |  |  |
| Alk |  |  |  |  |  |  |  |  |  |  |  |  |
| pH |  |  |  |  |  |  |  |  |  |  |  |  |
| Turb |  |  |  |  |  |  |  |  |  |  |  |  |
| TDS |  |  |  |  |  |  |  |  |  |  |  |  |
| T.H |  |  |  |  |  |  |  |  |  |  |  |  |
| E.C |  |  |  |  |  |  |  |  |  |  |  |  |
| Cl^-^ |  |  |  |  |  |  |  |  |  |  |  |  |
| NO_3_- |  |  |  |  |  |  |  |  |  |  |  |  |
| PO_4_- |  |  |  |  |  |  |  |  |  |  |  |  |

- **Author’s response:** According to the suggestion of hon’ble reviewer, table 2 has been re-arranged in the revised manuscript (Line no. 344-346).
